# Procalcitonin-Guided Antibiotics after Surgery for Peritonitis: A Randomized Controlled Study

**DOI:** 10.1155/2017/3457614

**Published:** 2017-05-16

**Authors:** Juliette C. Slieker, Steve Aellen, Philippe Eggimann, Valentine Guarnero, Markus Schäfer, Nicolas Demartines

**Affiliations:** ^1^Department of Visceral Surgery, Centre Hospitalier Universitaire Vaudois, Lausanne, Switzerland; ^2^Department of Surgery, Hôpital du Valais, Sion, Switzerland; ^3^Department of Intensive Care Medicine, Centre Hospitalier Universitaire Vaudois, Lausanne, Switzerland

## Abstract

**Background:**

Serum procalcitonin (PCT) is a useful biomarker to tailor the duration of antibiotics in respiratory infections. The objective of this study was to determine whether PCT levels could tailor postoperative antibiotic therapy in patients operated for peritonitis.

**Method:**

Patients with peritonitis were randomized postoperatively. The control group received antibiotics for a defined duration according to institutional guidelines. In the study group, antibiotics were stopped based on serum PCT levels. Patients were stratified into three categories: (1) gastrointestinal perforation, (2) perforated appendicitis, and (3) postoperative complication. Primary outcome was duration of antibiotics.

**Results:**

We included 162 patients; 83 and 79 patients in the control group and study group, respectively. In the subgroup of patients with peritonitis due to gastrointestinal perforation, we found 7 days of antibiotics in the PCT group versus 10 days in the control group (*p* value 0.065). There was no difference in infectious complications, mortality, median length of hospital stay, and necessity to restart antibiotics.

**Conclusion:**

No significant differences were found in duration of antibiotics when applying PCT guidance. However, in the subgroup of primary perforation of the gastrointestinal tract, there was a difference in duration of antibiotics in favor of the PCT group without obtaining significance, as the study was not powered for subgroup analysis. Further studies including only this subgroup should be performed.

## 1. Introduction

Successful management of peritonitis due to perforation of the gastrointestinal tract remains challenging, even in the era of modern surgery. Incidences of infectious and noninfectious complications remain high, as is the mortality rate [1]. Rapid diagnosis, efficient surgical intervention, and potent antibiotics are the most relevant cornerstones of any treatment algorithm. Perioperative broad spectrum antibiotics are commonly used, allowing clearance of aerobic and anaerobic bacteria. While antibiotics are empirically targeted at the usual digestive flora, there is poor consensus on the duration of antibiotic treatment, and most institutions have adopted their own guidelines. Nevertheless, appropriate shortening of the treatment duration may be a crucial aspect limiting antibiotic resistance, costs, duration of hospital stay, and improving patient's outcomes [2].

The use of biomarkers to assess the treatment efficacy on infections is commonly known, but a specific parameter to monitor the duration of antibiotics has been lacking. Procalcitonin (PCT), a precursor of calcitonin, is amplified as part of the physical response to bacterial infections [3, 4]. In animal models as well as in clinical settings, the correlation of PCT and the severity of sepsis have been shown to be superior to other biological markers, such as C-reactive protein, interleukin-1, or interleukin-6 [4–6]. Furthermore, there is solid evidence that the treatment of community-acquired pneumonia and ventilator-associated pneumonia can effectively be monitored by repeated measurement of serum PCT levels, and the duration of antibiotics can significantly be shortened [7–11]. Therefore, we hypothesized that the duration of antibiotic treatments based on current guidelines represents an “overtreatment,” and a tailored approach could shorten antibiotic therapy without increasing infectious complications in patients with gastrointestinal perforations.

The aim of this study was to assess the usefulness of PCT to guide the duration of antibiotic use in surgical patients with peritonitis due to gastrointestinal perforation.

## 2. Patients and Methods

### 2.1. Setting and Study Population

The current study is a prospective randomized trial performed at a university hospital. The duration of antibiotic therapy in patients treated according to institution guidelines with defined antibiotic duration (*control group*, Table 1) was compared to patients in whom antibiotic treatment was guided by serum PCT levels (*procalcitonin (PCT) group*). The study protocol was approved by the local ethical committee, registered (www.clinicaltrials.gov, NCT01155739), and all patients gave written informed consent.

### 2.2. Inclusion Criteria

All patients > 18 years who underwent abdominal surgery and with an established diagnosis of peritonitis were potentially eligible. All types of peritonitis caused by gastrointestinal perforations were included (gastric, purulent, fecal, or fibrinous content) either restricted to one quadrant or generalized to all four quadrants. After inclusion into the study, patients were stratified into three categories based upon the diagnosis: (1) peritonitis related to a gastrointestinal perforation (e.g., diverticulitis, perforated gastric or duodenal ulcer, intestinal ischemia, iatrogenous perforation, intestinal perforation in trauma patients, and perforated malignancy), (2) peritonitis due to perforated appendicitis, and (3) peritonitis due to a postoperative complication.

### 2.3. Exclusion Criteria

Patients with any kind of immunosuppression (e.g., long-term corticosteroids, chemotherapy, organ transplantation, and HIV with <200 CD4 cells), patients with medullary cancer of the thyroid or severe hepatocellular insufficiency (high procalcitonin values), and spontaneous peritonitis in patients with ascites were excluded. In addition, patients who refused to sign informed consent were not included.

### 2.4. Study Intervention and Randomization

Inclusion of patients was done postoperatively, after the diagnosis of peritonitis was established and its origin determined by surgery. Patients were randomly assigned to the control or PCT group through sealed, opaque envelopes. There was no stratification of randomization based on the three subgroups. Randomization was blinded for the patient, while physicians were aware of the respective group. In both groups, empiric antibiotic therapy was started preoperatively or at induction of anesthesia, and serum PCT levels were determined at the day of surgery or postoperative day 1. In the control group, duration of antibiotic application was according to institutional guidelines (Table 1), but of note, duration was prolonged if clinically indicated, for example, in case of persisting fever, increasing infectious laboratory values, or infectious complications (abscess, positive blood cultures).

In the PCT group, antibiotics were continued until postoperative day 3, without consideration of the respective serum PCT levels. On the third postoperative day, serum PCT values were measured and antibiotics were stopped if serum PCT values < 1 *μ*g/l; if values were >1 *μ*g/l, antibiotics were continued. Serum PCT values were determined every following 48 h, and in case of serum PCT values < 0.25 *μ*g/l or a decrease of 80% compared to the baseline value, antibiotics were then stopped. The daily physical examination of the patient was taken into account when stopping the antibiotics. In case of a clinical suspicion of an ongoing infection, antibiotics could be kept or reintroduced independently of the respective serum PCT levels or other diagnostic measures.

### 2.5. Measurement of PCT

Serum PCT measurements were performed with the Electrochemiluminescence immunoassay (ECLIA) on the automated Roche Elecsys® immunoassay analyzers and were obtained in approximately two hours.

### 2.6. Outcome Measures

The primary outcome measure was the duration of antibiotics in days. This includes the initial treatment, until discontinuation of antibiotics. If antibiotics were restarted, this was not included in the duration of antibiotics. Secondary outcome measures were (1) duration of hospitalization, (2) incidence of 30-day infectious complications, (3) 30-day mortality, and (4) incidence of reintroduction of antibiotics after having been ceased.

To compare groups, age, gender, comorbidities, ASA score, and Mannheim Peritonitis Index [12] were documented in all patients.

### 2.7. Statistical Analysis

The sample size was calculated based on a 33% reduction in antibiotic duration (10 days in control group, 6.7 days in study group, standard deviation 7.55 days). With a power of 80% and an *α*-error of 0.05 using a two-tailed test, 166 patients (83 per group) were necessary.

Categorical data are presented as numbers with percentages; numerical data are presented as medians with interquartile ranges. Univariate analysis was performed using a Fisher's exact test in case of categorical data, and a Mann–Whitney *U* test in case of numerical data. Data were analyzed as intention-to-treat. Statistical analysis was performed with SPSS version 22.

## 3. Results

**Figure 1:**
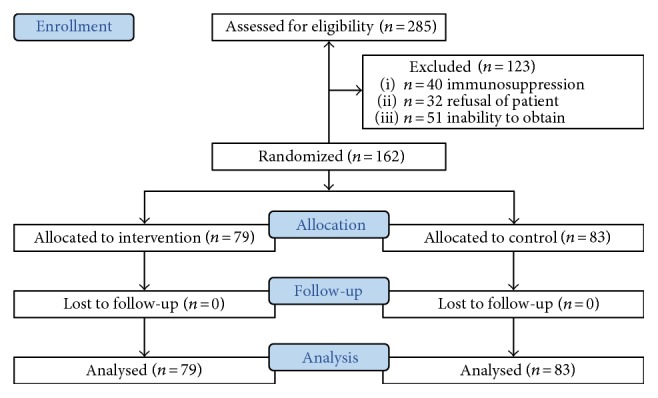
CONSORT diagram of included patients.

From June 2009 to September 2012, 162 patients with peritonitis were included and randomized between procalcitonin and control groups (CONSORT diagram Figure 1). Due to dropouts after initial inclusion, the power calculation was initially not obtained and the 10 last were included end 2013–beginning 2014 for this reason.

The baseline characteristics of all patients are shown in Table 2. There were 64 patients in the group with peritonitis related to a gastrointestinal perforation (group 1), 73 patients with peritonitis due to acute appendicitis (group 2), and 25 patients with peritonitis due to a postoperative complication (group 3).

The overall mortality rate was 4.3%, whereby these patients had a significant higher Mannheim Peritonitis Index (median 29 (range 23–34) versus median 17 (11.75–24), *p* value 0.005).

### 3.1. Primary Endpoint

When comparing all patients, there was no difference in duration of antibiotics between the PCT and control group (Table 3). The results for the subgroup analysis for patients with peritonitis related to a gastrointestinal perforation (group 1) separately show a trend towards a reduced duration of antibiotics in the PCT arm, without obtaining significance (median 7 versus 10 days, *p* value 0.065, Table 3).

### 3.2. Secondary Endpoints

There were no significant differences in infectious complications or death, as depicted in Table 4. Median duration of hospital stay was similar between both groups. Results of the secondary endpoints are shown for all patients together and for the subgroup analysis of group 1 separately since this group had a trend for significant difference for the primary endpoint.

**Figure 2:**
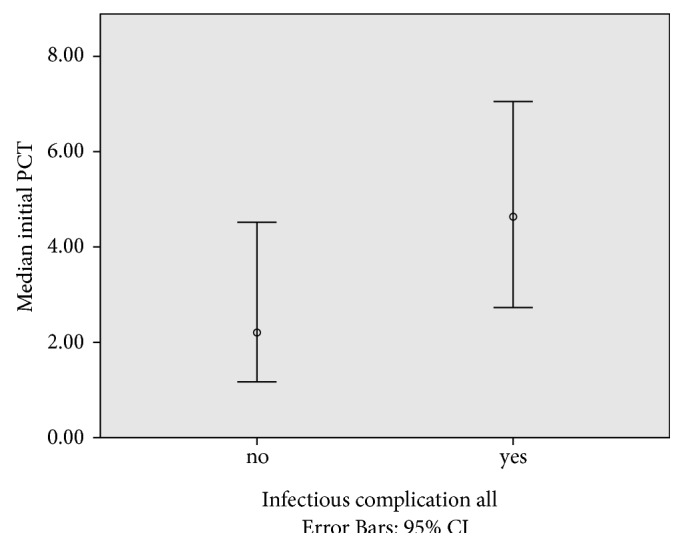
Relation initial PCT value and the occurring of postoperative infectious complications. *x*-axis show the group of patients with (yes) and without (no) an infectious postoperative complication. *y*-axis shows the median PCT value on day of surgery or postoperative day 1 in *μ*g/l (error bars 95% CI).

Figure 2 shows the relation of the initial level of procalcitonin to later complications. The median level of the initial PCT value (on day of surgery or the next day) in the group with no further complications was 2.2 *μ*g/l (interquartile range 0.5–7.5); whereas it was 4.6 *μ*g/l (interquartile range 1.3–14.7) in the group having one or more infectious complications in the postoperative phase. A Mann–Whitney *U* test shows that this difference is statistically different with a *p* value of 0.07.

**Figure 3:**
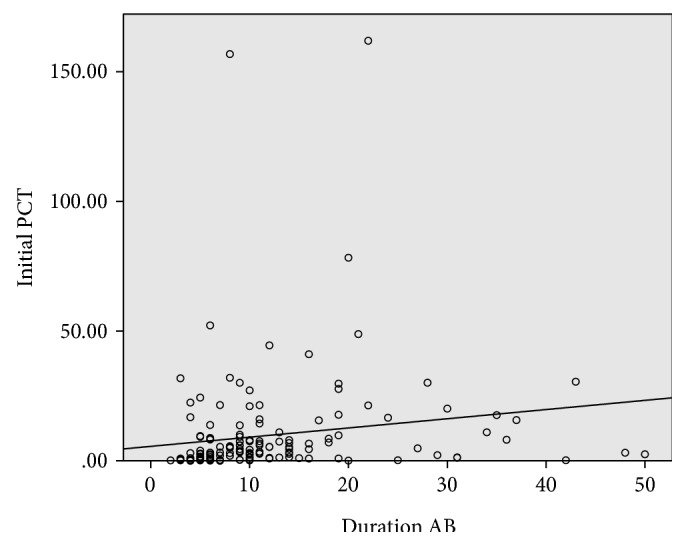
Relation initial PCT value and postoperative antibiotic therapy. Scatter plot of duration of antibiotic therapy in days on *x*-axis versus the median PCT value on day of surgery or postoperative day 1 in *μ*g/l. The Spearman's rank correlation coefficient *r* = 0.4, with a *p* value > 0.01.

Figure 3 shows the scatter plot of the initial level of procalcitonin to needed duration of antibiotic therapy. The Spearman's rank correlation coefficient is *r* = 0.4, with a *p* value > 0.01.

## 4. Discussion

This randomized controlled study assessed the role of a predefined algorithm using serum PCT levels to tailor the duration antibiotic therapies after surgery for peritonitis. To this end, 162 patient were randomized to receive either a PCT-guided therapy or a standard antibiotic treatment according to institutional guidelines. The main finding was that there were no differences of the duration of antibiotic treatment between both groups. In patients with an already short antibiotic treatment, for example, localized peritonitis after appendicitis, the algorithm failed to further shorten the duration of antibiotic use. Patients with peritonitis due to a postoperative complication (mostly anastomotic leakages) revealed the longest antibiotic therapies, but no reduction was achieved using the PCT algorithm. However, patients admitted to the hospital with peritonitis caused by primary gastrointestinal perforations were apt to the PCT algorithm, with a decreased median length of antibiotic treatment of three days, without obtaining statistical significance. This study was not powered for subgroup analysis; we speculate that no statistical significance was obtained due to insufficient power (type II error) due to a small patient subgroup. Further analysis of this subgroup showed no increased rate of infectious complications in the shorted antibiotics group compared to patients with a prolonged standard treatment time. The shortening of the antibiotic treatment duration may be a crucial aspect limiting antibiotic resistance, costs, duration of hospital stay, and improving patient's outcomes in this subgroup.

Interestingly, a higher initial value of PCT on postoperative day 0 or 1 seems to have a relation with a longer duration of antibiotic therapy and gives a higher risk of postoperative infectious complications.

This study was based on the positive results obtained with PCT guidance in pneumonia. Randomized controlled studies were set up in patients with suspicion of community-acquired pneumonia, and PCT values were used to initiate and to the duration of antibiotics [7, 8]. Initiation and duration was discouraged when finding PCT values less than 0.25 *μ*g/l and encouraged when greater than 0.25 *μ*g/l. These randomized studies showed that prescription of antibiotics on admission, total antibiotic exposure, and duration of antibiotic treatment was reduced in the PCT guidance group, compared with patients treated according to conventional guidelines. Corresponding results were obtained in ventilator-acquired pneumonia for the duration of antibiotics [9].

Our results are in line with the study by Huang et al. who performed a case-control study using a PCT algorithm to stop antibiotics in patients with secondary peritonitis [13]. They also showed a reduction of duration of antibiotic treatment by a median of 3 days, without observing an increased rate of adverse events. Another study on PCT after colorectal surgery found that PCT < 1.5 ng/ml on postoperative days 1 to 3 had a strong negative predictive value for systemic infectious complications [14].

There are some limitations of the current study that should be addressed. First, the duration of antibiotic treatment for peritonitis is not internationally standardized. Second, since an algorithm for discontinuation of antibiotics cannot completely replace clinical patient evaluations, treating physicians could violate the algorithm when clinically suspecting the need to continue antibiotic treatment. Last, the complete analysis is concordant with the prestudy power calculation but seems to include a too large variety of origins of peritonitis making the variance of duration of antibiotics too large for statistical significance. A subgroup analysis of a homogeneous group of patients approaches a level of significance without obtaining a statistical difference between groups. Since these are the results of a subgroup analysis, the lack of significance can be explained by a lower number of patients in the subgroup as anticipated.

In conclusion, a PCT algorithm could be used to guide antibiotic treatment in case of gastrointestinal perforation and allows a potential reduction in antibiotic duration. In particular, patients admitted with primary perforation of the GI tract may represent a suitable patient target group, whereas patients with an a priori limited time of antibiotic treatment do not benefit.

## Figures and Tables

**Table 1:** Institutional guidelines for duration of antibiotic therapy, based on the Surgical Infection Society guidelines [15–17].

Indication	Duration	First choice antibiotics
Perforated appendicitis with peritonitis	5 days	Amoxicillin/clavulanic acid Allergy: ciprofloxacin + metronidazole
Peritonitis due to gastrointestinal perforation	10 days	Amoxicillin/clavulanic acid. If severe infection: imipenem or piperacillin/tazobactam Allergy: ciprofloxacin + metronidazole
Peritonitis due to gastrointestinal perforation, acquired in-hospital	10 days	Imipenem or piperacillin/tazobactam Allergy: ciprofloxacin + metronidazole

**Table 2:** Baseline characteristics.

	Procalcitonin group (*n* = 79)	Control group (*n* = 83)	*p* value
*Median age (range), years*	56 (36–73)	57 (36–71)	0.776
*Males, n (%)*	46 (58.2%)	47 (57%)	0.875
*ASA 1-2, n (%)*	46 (58.2%)	60 (72.3%)	0.07
*ASA 3-4*	33 (41.8%)	23 (27.7%)	
*Comorbidities, n (%)*			
(i) Diabetes mellitus	4 (5.1%)	4 (4.8%)	1.00
(ii) Pulmonary	13 (16.5%)	7 (8.4%)	0.15
(iii) Cardiac	29 (36.7%)	34 (41%)	0.63
(iv) Renal dysfunction	9 (11.4%)	8 (9.6%)	0.80
*Needing ICU hospitalization, n (%)*	10 (12.7%)	10 (12.0%)	1.00
*Mannheim Peritonitis score (points)*			
(i) All	19 (11–25)	17 (12–26)	1.00
(ii) Subgroup 1	20 (16–26)	21 (15.5–27.5)	
(iii) Subgroup 2	15 (10.8–21.3)	15 (10–21)	
(iv) Subgroup 3	24.5 (17.8–29)	28 (21–38)	
*Peritonitis*			0.91
(i) Trouble exsudat	5 (6.3%)	7 (8.4%)	
(ii) Gastric/duodenal content	4 (5.1%)	5 (6.0%)	
(iii) Fibrinous	3 (3.8%)	3 (3.6%)	
(iv) Purulent	53 (67.1%)	52 (62.7%)	
(v) Fecal	14 (17.7%)	16 (19.3%)	
*Peritonitis quadrants*			0.73
1	33 (41.8%)	28 (33.7%)	
2	10 (12.7%)	14 (16.7%)	
3	3 (3.8%)	2 (2.4%)	
4	33 (41.8%)	39 (47.0%)	
*Group 1 peritonitis due to GI perforation*	31	33	0.95
(i) Diverticulitis	14	9	
(ii) Perforated ulcer	4	11	
(iii) Perforation stent/colonoscopy/biopsy	6	5	
(iv) Ischemia–necrosis	6	2	
(v) High-velocity trauma	0	2	
(vi) Perforated tumor	1	1	
(vii) Other	0	3	
*Group 2 peritonitis due to appendicitis*	34	39	0.61
*Group 3 postoperative peritonitis*	14	11	0.43
(i) Anastomotic leakage	6	5	

ASA: American Society of Anesthesiologists physical status classification system; ICU: intensive care unit.

**Table 3:** Primary outcome measure: duration of antibiotics (median with interquartile range, days).

Duration antibiotic treatment	Procalcitonin (*n* = 79)	Control (*n* = 83)	*p* value
(i) All patients	8 (5–16)	10 (6–12)	0.714
(ii) Subgroup 1: GI perforation	7 (5–12)	10 (8.5–12)	0.065
(iii) Subgroup 2: appendicitis	8 (5.5–13.5)	8 (5–11)	0.573
(iv) Subgroup 3: postoperative	18.5 (6.75–29.5)	13 (11–18)	0.403

**Table 4:** Secondary outcome measures.

	Procalcitonin		Control		*p* value	
	All (*n* = 79)	Subgroup 1 (*n* = 31)	All (*n* = 83)	Subgroup 1 (*n* = 33)	All	Subgroup 1
Duration of hospitalization, d	10 (5–24)	10 (6–24)	8 (4–16)	10 (6–20)	0.188	0.809
Reintroduction of antibiotics	6 (7.7%)	3 (10%)	5 (6.1%)	1 (3%)	0.762	0.340
Reoperation	24 (30.4%)	6 (19.4%)	20 (24.1%)	7 (21.1%)	0.383	1.00
Relavage abdominal cavity	10 (12.7%)	2 (6.5%)	7 (8.4%)	3 (9.1%)	0.447	1.00
Rehospitalization	5 (6.6%)	1 (3.6%)	5 (6.1%)	2 (6.1%)	1.00	1.00
Positive blood cultures	2 (2.5%)	0	3 (3.6%)	2 (6.1%)	1.00	0.493
Intra-abdominal abscess	14 (17.7%)	3 (9.7%)	14 (16.9%)	3 (9.1%)	1.00	1.00
Wound infection	17 (21.5%)	7 (22.6%)	15 (18.1%)	11 (33.3%)	0.694	0.410
Septic shock	4 (5.1%)	1 (3.2%)	4 (4.8%)	3 (9.1%)	1.00	0.614
Anastomotic leakage	3 (3.8%)	1 (3.2%)	3 (3.6%)	2 (6.1%)	1.00	1.00
Urinary infection	3 (3.8%)	2 (6.5%)	1 (1.2%)	0	0.358	0.231
Pneumonia	2 (2.5%)	0	4 (4.8%)	3 (9.1%)	0.682	0.239
Mortality	3 (3.8%)	3 (9.7%)	4 (4.9%)	3 (9.4%)	1.00	1.00

Duration of antibiotics and duration of hospitalization are medians with interquartile range (days). All other parameters are numbers with percentages.
